# Interpretability of Input Representations for Gait Classification in Patients after Total Hip Arthroplasty

**DOI:** 10.3390/s20164385

**Published:** 2020-08-06

**Authors:** Carlo Dindorf, Wolfgang Teufl, Bertram Taetz, Gabriele Bleser, Michael Fröhlich

**Affiliations:** 1 Department of Sports Science, Technische Universität Kaiserslautern, Erwin-Schrödinger-Str. 57, 67663 Kaiserslautern, Germany; michael.froehlich@sowi.uni-kl.de; 2 Junior Research Group wearHEALTH, Technische Universität Kaiserslautern, Gottlieb-Daimler-Str. 48, 67663 Kaiserslautern, Germany; teufl@cs.uni-kl.de (W.T.); taetz@cs.uni-kl.de (B.T.); bleser@cs.uni-kl.de (G.B.)

**Keywords:** explainable artificial intelligence, inertial measurement unit, machine learning, biomechanics, gait, total hip replacement

## Abstract

Many machine learning models show black box characteristics and, therefore, a lack of transparency, interpretability, and trustworthiness. This strongly limits their practical application in clinical contexts. For overcoming these limitations, Explainable Artificial Intelligence (XAI) has shown promising results. The current study examined the influence of different input representations on a trained model’s accuracy, interpretability, as well as clinical relevancy using XAI methods. The gait of 27 healthy subjects and 20 subjects after total hip arthroplasty (THA) was recorded with an inertial measurement unit (IMU)-based system. Three different input representations were used for classification. Local Interpretable Model-Agnostic Explanations (LIME) was used for model interpretation. The best accuracy was achieved with automatically extracted features (mean accuracy M_acc_ = 100%), followed by features based on simple descriptive statistics (M_acc_ = 97.38%) and waveform data (M_acc_ = 95.88%). Globally seen, sagittal movement of the hip, knee, and pelvis as well as transversal movement of the ankle were especially important for this specific classification task. The current work shows that the type of input representation crucially determines interpretability as well as clinical relevance. A combined approach using different forms of representations seems advantageous. The results might assist physicians and therapists finding and addressing individual pathologic gait patterns.

## 1. Introduction

Identification and discrimination of group differences are important aspects of biomechanical research [1,2]. With modern movement tracking systems, a huge amount of data is available (big data) [3,4]. The progressive development of motion analysis systems based on inertial measurement units (IMUs) contributes in particular to the generation of large amounts of data, because they make valid and reliable biomechanical data easily accessible [5]. This provides the potential to generate new knowledge and a better understanding of human biomechanics. However, classical inference-based statistical methods show limited capabilities in analyzing the emerging—often complex and multivariate—amounts of data and, thus, machine learning models have gained importance [3,4,6].

Many studies have obtained promising results for the classification of pathological movements (e.g., hip osteoarthritis [7], after stroke [8], and Parkinson’s Disease [9]). However, many of the machine learning models show black box characteristics and a lack of transparency [10]. For example, this does not comply with the requirements of the European General Data Protection Regulation (GDPR, EU 2016/679) [11] and strongly limits practical application in clinical contexts. In order to improve interpretability, transparency, and clinical relevancy, the models themselves (model interpretability) as well as the practical usefulness and interpretability of the input variables used for modeling (feature interpretability) must be taken into account.

The input representation plays an important role in classification accuracy [12], as well as in interpretability, clinical relevancy, and comparability with previous research. Different feature extraction approaches can be found in the literature:

(i) Many studies have used simple descriptive statistics of the gait waveforms such as peak values, range of motion, or respective side differences [13,14]. They are straight forward to interpret and are often mentioned in the literature for describing gait characteristics. However, it is unclear if important information would be a priori discarded, and model performance is consequently negatively affected. A further limitation is the dependence on expert or prior knowledge.
(ii) An alternative approach, which is independent of prior knowledge, is the use of entire concatenated waveforms as input features [7,15,16]. This allows for an interpretation of group differences through the determination of important areas of the waveforms. However, it is unclear if this shows better discriminative power compared with the abovementioned extracted statistical features and, therefore, enhances classification performance. Correlations and redundancy of the inputs may further be problematic.
(iii) Lastly, automated feature extraction using a vast amount of possibly meaningful statistics can be applied [17]. Feature extraction algorithms such as tsfresh [18] or featuretools [19] can be used for this. However, the extracted features are often nested, complex, and hard to interpret, therefore showing limited comparability with the literature, which results in questionable clinical relevance.

Dimensionality reduction methods can be applied before classification to compress the data onto a new feature space, with the aim of capturing as much variance as possible of the original data [15,20]. Nevertheless, this makes interpretation harder because the components of the new feature space must be interpreted first. For all mentioned approaches, it should be noted that feature selection may further improve a model’s accuracy, reduce computing power, prevent overfitting, and improve interpretability [21].

Regarding model interpretability, on the one hand, complex machine learning models (e.g., deep neural networks) often achieve more accurate results compared with simpler models (e.g., decision trees). On the other hand, complexity leads to reduced transparency and interpretability (trade-off between model accuracy and interpretability) [22,23], which results in the black box characteristic of many models [10]. For the user, it is therefore hard to trust in the model and its decision because it is opaque as to what the model has really learned and why it makes certain decisions [24]. These factors currently limit practical application in clinical contexts [25].

Explainable Artificial Intelligence (XAI) has gained great interest in recent years and offers methods for increasing the transparency and trustworthiness of black box models [10]. Local Interpretable Model-Agnostic Explanations (LIME) [26], SHapley Additive exPlanations (SHAP) [27], and Deep Learning Important FeaTures (DeepLIFT) [28] should be mentioned as prominent interpretation tools. For example, LIME performs an approximation of a single prediction of a black box model with a simpler interpretable model to explain how a black box model makes a single prediction [26]. Initial studies applying XAI methods with clinical data have produced promising results [16]. Therefore, XAI methods seem promising for making machine learning models more useable in practical clinical applications.

The application of XAI methods in the context of biomechanical data analysis is a young field of research. Practical applications in clinical contexts are still very rare. There is need for research in order to assess potentials, show limitations, and identify further research directions in the biomechanical and clinical domain. As a step towards practical clinical application, the present work focuses on the input representation. Different kinds of input representations take different perspectives on the data and provide different insights. Yet, they all show benefits and limitations regarding model accuracy, interpretability, and clinical relevancy. The application of XAI methods based on different input representations could, therefore, lead to new insights and an even better understanding of the data. To the best of our knowledge, no similar comparison has been performed so far. Therefore, we wanted to check if the application of XAI methods on models, trained on different input representations, leads to congruent results and provides, taking them all together, better interpretability. For this reason, we used a highly relevant use case example and compared the gait kinematics, measured by means of an IMU system, of patients after total hip arthroplasty (THA) (most important surgery for the treatment of degenerative hip osteoarthritis [29]) with that of a group of healthy subjects. 

## 2. Materials and Methods

### 2.1. Subjects, Data Acquisition, and Data Preprocessing

For the present study, the IMU-based gait data of a healthy sample from [30] and a sample of patients after THA from [13] were employed. The studies were approved by the ethical committee of the Technische Universität Kaiserslautern and the Universität Paderborn and met the criteria of the Declaration of Helsinki. After receiving all relevant study information, the participants signed an informed consent form for the study, including permission to publish the data.

In the mentioned studies, 27 healthy subjects (14 females, 13 males; age: 24.63 ± 2.80 years; weight: 70.44 ± 12.56 kg; height: 1.76 ± 0.09 m) and 20 subjects approximately 2 weeks after THA (13 females, 7 males; age: 57.79 ± 7.41 years; weight: 83.89 ± 17.22 kg; height: 1.73 ± 0.08 m) performed a 6 min walking test. The accelerometer and gyroscope raw data during the gait were recorded by means of seven MTw Awinda IMUs (Xsens Technologies BV, Enschede, The Netherlands) attached to the segments of the lower extremities according to [13].

The IMU raw data were then processed using a recently developed sensor fusion algorithm based on an extended Kalman filter approach [31,32]. Using this algorithm, relative segment orientations were estimated exploiting the knowledge of a biomechanical model (i.e., segment lengths, joint centers, virtual anatomical landmarks, and an IMU to segment calibration). From this, it was then possible to interpret these angles as anatomically meaningful joint angles and further estimate gait-specific events (i.e., initial contact and terminal contact), and calculate based on those spatiotemporal parameters. The event detection and the estimation of the joint angles of the lower body using the mentioned algorithm were validated in recent publications [30,33,34].

Consequently, the following parameters were calculated based on the IMU data: the hip, knee, and ankle joint angle waveforms as well as the global pelvic motion in the sagittal, frontal, and transversal plane.

The joint angle waveforms of all subjects were divided into gait cycles (GCs) using the initial contact information. The initial contact was detected using a kinematics-based approach according to [34]. The gait cycles were then checked for outliers using the mean gait cycle duration (i.e., the stride time) of a subject ± 2 times standard deviation. Gait cycles displaying a duration higher or lower than these thresholds were excluded from the following evaluation. The remaining gait cycles of all subjects were normalized to 100-time steps using cubic spline interpolation. Twenty gait cycles were extracted for every subject. The original sidewise (left, right) consideration was transformed into a differentiation between affected and unaffected sides.

Three different forms of input representations were calculated and used as input vectors: entire waveforms (V_waves), discrete features based on simple descriptive statistics (V_simple), and automatically extracted features (V_tsfresh) (see Table 1).

To analyze the influence of different data preprocessing, training and test data were standardized based on the respective training set, with three different scaling approaches:

- without data scaling,
- removal of the mean and scaling to unit variance (StandardScaler),
- scaling to a feature range between 0 and 1 (MinMaxScaler).

The processing of the IMU raw data and the calculation of the joint angle waveforms were conducted in C++. The segmentation of the joint angle waveforms as well as the outlier detection were conducted in Matlab 2019b (Mathworks, Inc., Natick, MA, USA). Further calculations were performed in Python (Python Software Foundation, Wilmington, DE, USA).

### 2.2. Model Training and Classification

The following procedure was applied for all input vectors: fivefold cross-validation was performed with each test set consisting of the gait cycles respective to the extracted features of four subjects of each class (8 subjects in total with 8 × 20 GCs). Every subject of the THA group was used once for testing. Due to imbalanced class distribution in the training set, synthetic minority oversampling [37] was for each fold performed on the respective training set. Random Forest (RF), linear Support Vector Machine (SVM), SVM with radial basis function kernel (rbf), and a neural network (multilayer perceptron, MLP) were applied for classification with the standard parameters of Scikit-learn [38].

### 2.3. Model Interpretation

An a priori specification of the best performing classification algorithm for a certain task is in most cases not possible. For practical application, interpretation tools should, therefore, be generally applicable (model agnostic) and not dependent on a specific algorithm (model specific). Further, subjects show individual gait characteristics [39,40]. In the context of personalized medical treatment, it becomes, therefore, especially important to understand why a model made a specific decision for a single instance/GC or for a single subject, respectively. Therefore, local interpretability gains importance.

For that reason, the model-agnostic interpretation tool LIME [26] was used for model interpretation. To explain a black box model, LIME performs an approximation of a single prediction of a black box model with a simpler interpretable model (e.g., decision tree, linear model). The simpler model will probably not perform a globally faithful approximation of the complex model but will perform well locally. Therefore, LIME is based on local interpretations of single instances of interest. To explain a single prediction of a black box model, the instance of interest is chosen and data points around it are generated through perturbation. These data points are predicted with the black box model and weighted by their proximity to the selected instance. Finally, a simpler model is learned on the weighted data points and used for explaining the prediction [26].

For the special case of serial data (gait waveforms), LIME for Time was developed [41]. It is based on the idea of using LIME in the context of image data. For image data, Superpixels (connected pixels of one color) are used for data variation because variations of individual pixels would hardly change a model’s prediction. As an equivalent to Superpixels, LIME For Time performs data variation of parts of a series by replacing them, for example, by noise or the entire mean of the series. The approach makes it possible to identify areas of serial data that are important for the classifier in its prediction. The algorithm was applied for interpretation of the concatenated waveforms (V waves). Total mean was used for perturbation.

For each input vector, the best performing model was used for interpretation with LIME. Local interpretations are presented as exemplars. To determine if effects due to group differences were important for class selections across multiple instances, global interpretations are presented (indication of generality). They were calculated by mean aggregation of the absolute weight values of the local results (5 GCs of each subject to reduce computing power) of the LIME algorithm (see, e.g., [42]). Statistical Parameter Mapping (SPM) [43] was used to verify the results from a statistical perspective.

## 3. Results

### 3.1. Classification Results

**Figure 1:**
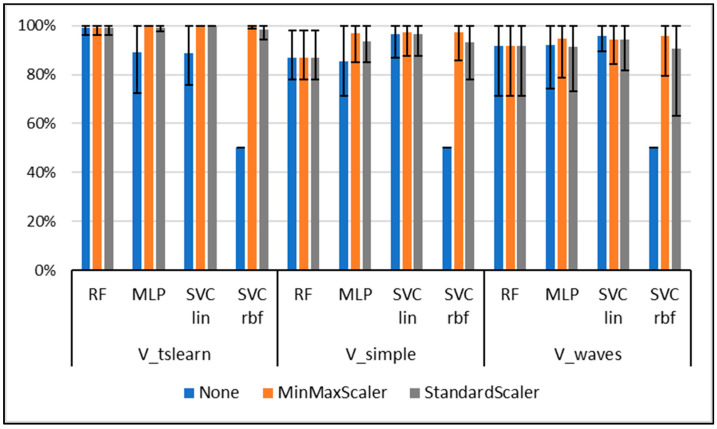
Classification accuracy (mean, min, max value) for the different input vectors, classification algorithms, and normalization approaches over the 5-fold cross-validation. RF = Random Forest; MLP = multilayer perceptron; SVM lin = Support Vector Machine with linear kernel; SVM rbf = Support Vector Machine with radial basis function kernel.

Classification results are presented in Figure 1. The overall best accuracy was obtained for the use of the V_tsfresh input vector, SVM linear (MinMaxScaler, StandardScaler), and MLP (MinMaxScaler) (mean accuracy M_acc_ = 100%). The best result for the waveform data V_waves was obtained with SVM linear without normalization (M_acc_ = 95.88%). SVM linear with MinMaxScaler performed best for the gait-specific data (M_acc_ = 97.38%). The best performing models are used in the following subsections for interpretation.

### 3.2. Model Interpretation Based on Waveforms

**Figure 2:**
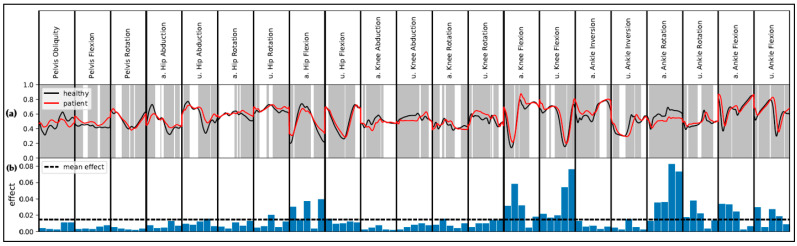
Global results for the use of the waveform data (V_waves). For visualization reasons, data are separately scaled to a range from 0 to 1 for each variable. (**a**) Mean movements for healthy subjects and patients after total hip arthroplasty (THA). The grey areas indicate statistical difference (alpha = 0.05) according to Statistical Parameter Mapping. (**b**) Aggregated Local Interpretable Model-Agnostic Explanations (LIME) results as mean absolute effect. Abbreviations: a. = affected side; u. = unaffected side.

The global results for the comparison of patients and healthy subjects based on the concatenated waveforms (V waves) are presented in Figure 2. SPM indicates statistical group differences for most of the movements (*p* < 0.05). LIME indicates a high global effect for slices representing the ankle rotation as well as knee and hip movement in the sagittal plane. The ankle rotation of the affected side, especially during initial contact and midstance, plays an important role. For the knee joint of the affected as well as unaffected side, the important slices represent the maximal flexion and the maximal extension. Maximal flexion and maximal extension are further relevant for the hip movement in the sagittal plane of the affected side. Group differences for the respective slices are all significant.

**Figure 3:**
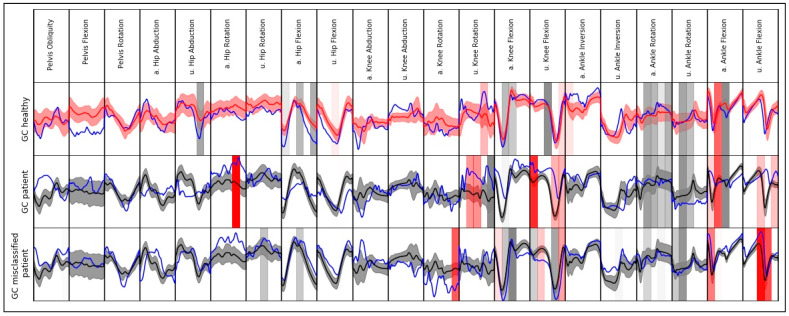
Exemplary local model interpretation for single gait cycle (GC) (instance) of a patient with THA, a healthy subject, and a GC of a patient classified as healthy (blue lines). The instances are plotted against the mean value and standard deviation of the other class (black = healthy, red = patient). For better visualization, the top 20 slices with the highest absolute effect are displayed for each instance (grey vertical span = effect towards class of healthy subjects, red vertical span = effect towards class of patients with THA). The color saturation indicates the effect size. For visualization reasons, data are separately scaled to a range from 0 to 1 for each variable. Abbreviations: a. = affected side; u. = unaffected side.

Model explanations for single instances (gait cycles) regarding the waveform data are presented in Figure 3. It is noticeable that for both correctly classified GCs, few slices indicate an effect towards the other class. Additionally, one misclassified GC of a patient is shown. Most of the slices with the highest effect indicate an effect towards the class of healthy subjects (e.g., ankle rotation, knee flexion). Similar movement patterns compared to healthy subjects are noticeable for the respective slices.

### 3.3. Model Interpretation: Discrete Features 

**Figure 4:**
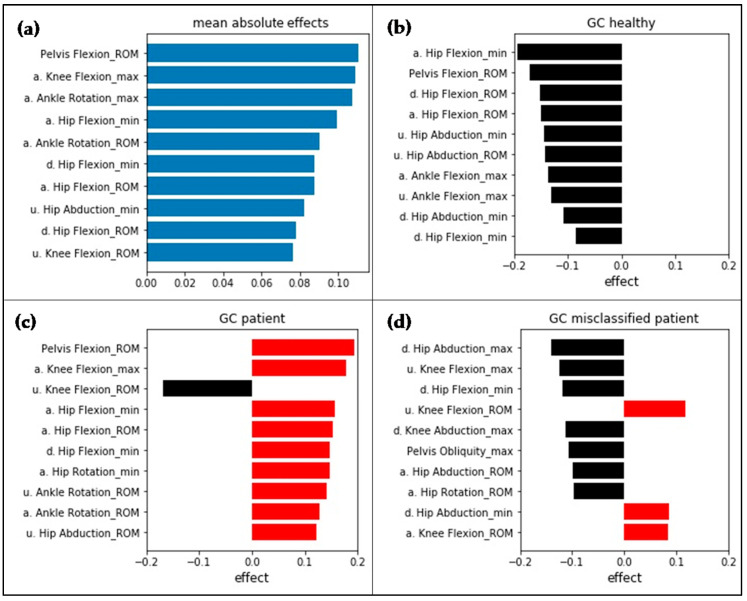
(**a**) Global LIME results as mean absolute effects. The analyzed instances (**b**–**d**) correspond to the instances displayed in Figure 3. Negative values indicate an effect towards the class of healthy subjects (black), positive effects towards the class of patients after THA (red). Feature abbreviations: a. = affected side; u. = unaffected side; d. = difference affected, unaffected side.

LIME results for the use of the V simple input vector are presented in Figure 4. The analyzed instances correspond to the interpreted waveform instances displayed in Figure 3. Even though different input features were used, the model performed the same misclassification. The features with the highest effect globally seen are based on hip, knee, and pelvic sagittal motion and ankle rotation in the transversal plane.

**Figure 5:**
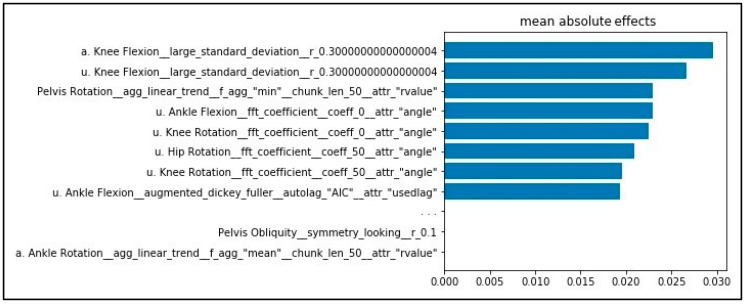
Mean absolute effects for the V_tsfresh input vector determined using LIME. Results are based on SVM linear with MinMaxScaler. Feature labels are according to the automated feature extraction algorithm tsfresh [18].

The mean absolute effects for the use of the V_tsfresh input vector are shown in Figure 5. Features based on knee and ankle sagittal movement as well as hip, knee, and pelvic transversal movement show the highest effect. For calculation of the features with the highest effect, the operation “large_standard_deviation” was used. The result of the operation is a Boolean variable, denoting if the standard deviation of the series (in this case, knee flexion) is higher than “r” times the range of motion (ROM).

## 4. Discussion

Very good results regarding accuracy were obtained for the classification of patients with THA and healthy subjects. Three different input vectors were used. The best classification results were obtained when using automatically extracted features (V tslearn). However, with the use of simple descriptive statistics (V simple) or the waveform data (V waves), very good classification performances with only a slight reduction in accuracy were obtained. It can be assumed, in line with the previous study [13], that even simple descriptive statistics have high discriminative power, appropriately map the classes, and, therefore, slightly outperform the pure use of the waveforms.

In line with other works [8], we can demonstrate the superior performance of linear SVM in the context of gait classification. In most cases, linear SVM showed the best results and outperformed the more complex and computationally expensive models (e.g., MLP). The main focus of the current work was on interpretability; therefore, no extensive parameter tuning of the models was performed. It might be possible to obtain the same or even better results using the more complex models. However, the cost would be an extensive and time-consuming parameter tuning.

With the use of the model agnostic method LIME, it was possible to gain insight into how the models made their decisions and to increase interpretability. Through the selection of a maximum number of features to be displayed by LIME, it is possible to provide different interpretation levels for different contexts. Using complex features (V tsfresh) leads to slightly higher performance with the cost of interpretability and comparability with previous research. The reason for this is that the operations used for feature calculation often make it difficult to attribute class differences to the direct movements because they describe the original movements on a more abstract level compared with simpler and, in a biomechanical context, more commonly used descriptive statistics. Therefore, their usage in clinical contexts is questionable. Thus, the following interpretation mainly focuses on the input representations V waves and V simple. 

To evaluate the validity/plausibility of the effects and the identified group differences, three aspects are addressed as follows: (i) asymmetric gait patterns are often prevalent after THA [15,44]. Not only are the operated joint and surrounding structures affected but also the contralateral side [36]. However, the main difference of the gait characteristics between healthy subjects and patients after THA should be seen for the affected side. LIME results should therefore generally emphasize an effect for features regarding the affected side. For both input vectors, most of the time, this holds true. Yet, some features with the highest effect map gait differences for the unaffected side (e.g., V_simple: unaffected hip abduction) or for both affected and unaffected sides (e.g., waveform data: unaffected/affected knee flexion/extension). A possible reason for this might be that the trained model compares the affected and unaffected sides. Regarding the sample of subjects, age-related differences in gait, which possibly led to a group separation with regard to the unaffected side (see, e.g., classification young, old [45]), should not go unnoticed and possibly have further influence.

(ii) Another aspect which speaks for the validity of the results is that most results are congruent between V_tsfresh and V_wave. For both input vectors, ankle rotation as well as sagittal knee and hip movement plays an important role. Nevertheless, pelvic flexion (ROM) only shows a high effect for the use of V_tsfresh. Therefore, application of different input representations can provide new insights and show effects, which were possibly not detected with only the use of a single input vector.

Using only the waveform data, interactions between different slices are opaque. For example, maximal flexion and maximal extension of hip movement in the sagittal plane is highly relevant regarding the waveform data. Looking at V_tsfresh, the ROM of the hip movement in the sagittal plane shows a high effect. This might indicate that the slices mapping maximal flexion and maximal extension interact. Comparing the results for different input vectors might, therefore, be useful for a better understanding of possible interactions.

(iii) Finally, LIME results are discussed taking into account previous research. Overall, a ground truth of the automatically determined explanations is missing, and it therefore becomes more difficult to evaluate if the results are meaningful and appropriately map gait characteristics. In the literature, gait patterns of patients with THA are mostly described using simple descriptive statistics comparable with the V_simple input vector [36,46,47]. Consequently, it is harder to directly compare the effects respective to the determined relevant regions regarding the waveform data (V wave) with previous works. Further, it is not possible to evaluate all our findings with previous research because the literature often focuses on a few gait characteristics to describe group differences. In agreement with our findings, previous research reports a reduced ROM for knee and hip movement of the operated side in the sagittal plane compared with healthy subjects [36,44,46,47,48]. Further, altered postoperative ankle rotation [46] and increased sagittal pelvic movement compared with healthy subjects was found [46,47].

Regarding the current state of research, there are no objective criteria to evaluate interpretability [42]. Subjective ratings from end-users or task performance might be possible ways for evaluation [22]. Regarding interpretability and the goal of making machine learning applicable in practical clinical contexts, it is important to make a distinction between the interpretability of the model itself and clinical interpretability. Interpretability of a model describes the degree to which humans can understand a decision from a model by its causes and, hence, why a model made a certain decision [49]. The usage of XAI tools in this study made it possible to understand why a model made a certain decision through revealing the effects for features or areas of waveforms which were important for a certain prediction. However, with this alone, it is unclear if the identified effects are interpretable, relevant, and usable in clinical contexts. In this respect, the current work emphasizes the importance of the input representation because it highly influences the interpretability and usability in clinical contexts. In the current case, automatically extracted features show no significantly better classification accuracy, which could possibly justify a loss of interpretability. Consequently, waveform data and simple descriptive statistics should be used for practical applications because effects can be traced back to variables that are mentioned in the literature and already used in clinical contexts for describing biomechanical differences. 

In cases where classification is harder, a combination of expert-knowledge-based features extended by the best performing automated extracted features might be an appropriate compromise to increase classification performance and ensure best possible clinical interpretability. In settings where only classification performance without the need for biomechanical interpretability is important, automated feature extraction approaches can be suggested.

Simple metrics are often used for describing pathologic movements (e.g., symmetry index [48]) in clinical contexts because the full consideration of all movements is too complex for human beings. Further, many clinical decisions are influenced by expert knowledge and the experiences of the physicians and therapists. As the current study demonstrates, the benefits of data-driven approaches based on machine learning and XAI are their ability to take into account the full complexity of the motion data, and they therefore may provide objective orientations and assistance for physicians in their decisions. Further, through focusing on local model interpretability, they are able to take into account the individual gait characteristics that were important for classification and class discrimination and, therefore, play an important role in the context of personalized medicine.

Local interpretability additionally helps one to understand why a model wrongly classified single instances. As previously presented as an example, a GC of a patient was wrongly classified as a GC of a healthy person. A possible explanation is that the subject showed fewer pathologic gait patterns compared with the remaining patients and was therefore classified as a healthy person. In this regard, the algorithm could provide information about the rehabilitation status of a patient and an objective orientation for physicians and therapists. However, it cannot be excluded that the model was not able to handle and correctly classify the instance, because the regarded instance was an outlier. In this regard, the usage of automatic systems is dependent on experts, because in such cases, expert knowledge and experience is crucial for the right decision-making.

The current work emphasizes the importance of data preprocessing for a model’s accuracy and interpretability and shows that it is not possible to give general recommendations. In the present case, the best results were obtained without scaling for the use of gait waveform data. This might also be promising for interpretation because features can be directly interpreted without the need of scaling the data back to its original representation.

Another important aspect is data aggregation. In the present case, in line with various other studies [50,51], single gait cycles were used for input feature calculation. However, in the literature, averaged waveforms were also reported for gait classification [20,52], which might be useful for elimination of the intraindividual variance between different gait cycles. Depending on the requirements in possible fields of application, this might be an alternative approach. Various other methods should not go unnoticed for dealing with imbalanced data (see, e.g., [53]) and should be further considered and compared in future works. 

Previous research showed that IMU-based systems show higher errors for movements in the transversal plane [54]. Therefore, it is uncertain if the findings for transversal movements are meaningful and interpretable. The exclusion of transverse movements due to great inaccuracies when using IMU-based systems might, therefore, be considered in future works.

As noted previously, there are limitations regarding the sample of subjects. In particular, the large age and weight difference between the groups of healthy subjects and patients after THA is striking. At this point, it cannot be excluded that the corresponding effects influenced the classification. In consecutive studies, the analysis should be repeated with a matched group of healthy subjects. Further, it worth mentioning the possible strong correlations between the features which are a priori present and lead to redundancies in the different input vectors. To reduce the impact of redundant features, application of the minimum redundancy maximum relevance (mRMR) filter [15,55] might be a promising approach.

The present work used the LIME algorithm and checked if its application would lead to coherent results from different input representations. The utility and generalizability of the mentioned methodology should be evaluated through application on data of different subjects and groups of patients. Moreover, future works should compare different explanation methods (e.g., LIME, SHAP, DeepLIFT) and check whether results are consistent. 

## 5. Conclusions

XAI is promising for making the decisions of machine learning models more transparent, interpretable, and, therefore, more trustworthy. It is, therefore, a promising step towards the practical applicability of machine learning in clinical contexts. However, the research is still very young in this domain. Before practical clinical application, further research is necessary. The current study shows that the type of input representation crucially determines interpretability as well as clinical relevancy. A combined approach using different forms of representations seems advantageous, because it can provide a better understanding of the underlying group differences and discriminative effects that were important for classification. Based on the current findings, waveform data as well as features based on simple descriptive statistics can be suggested. In the context of personalized medicine, XAI approaches focusing on local interpretability enable the identification of individual gait characteristics, which are important for classification and class discrimination. Thus, the results might assist physicians and therapists in finding and addressing individual pathologic gait patterns by offering an objective orientation.

## Figures and Tables

**Table 1:** Input vectors used for modeling. Twenty gait cycles (GCs) per subject were extracted. To reduce the number of irrelevant features of V_simple and V_tsfresh, the fresh-algorithm (FeatuRe Extraction based on Scalable Hypothesis tests) [18] was applied as a filter. ROM = range of motion

Abbreviation	Description	Size (GC × Feature)
V_waves	Concatenated time-normalized GC for the measured variables.	940 × 2100
V_simple	Calculated features based on simple descriptive statistics which are commonly mentioned in the literature [35,36]. Maxima, minima, and ROM for every variable as well as the difference between affected and unaffected sides for the respective variables were calculated.	940 × 74
V_tsfresh	Automated feature extraction with the tsfresh algorithm [18].	940 × 8349
